# Practical considerations for monitoring patients with familial Mediterranean fever: review of evidence and expert opinion

**DOI:** 10.3389/fimmu.2026.1857052

**Published:** 2026-07-28

**Authors:** Jasmin B. Kuemmerle-Deschner, Abdurrahman Tufan, Luca Cantarini, Andreas Clemens, Albert Schuen, Ruby Haviv

**Affiliations:** 1 autoinflammation reference center Tübingen (arcT), Division of Paediatric Rheumatology, Department of Paediatrics, University Hospital Tübingen, Tübingen, Germany; 2 Division of Rheumatology, Department of Internal Medicine, Gazi University Faculty of Medicine, Ankara, Türkiye; 3 Department of Medical Sciences, Surgery and Neurosciences, Rheumatology Unit, University of Siena and Azienda Ospedaliero-Universitaria Senese [European Reference Network (ERN) for Rare Immunodeficiency, Autoinflammatory and Autoimmune Diseases (RITA) Center], Siena, Italy; 4 Novartis Pharma AG, Basel, Switzerland; 5 Department of Pediatrics, Pediatric Rheumatology Unit, Meir Medical Center, Kfar Saba, and School of Medicine, Faculty of Medical and Health Sciences, Tel Aviv University, Tel Aviv, Israel

**Keywords:** biomarkers, disease activity, familial Mediterranean fever, management, patient monitoring, treat-to-target

## Abstract

**Introduction:**

Effective monitoring is critical in familial Mediterranean fever (FMF), to guide a treat-to-target approach to management and to prevent long-term complications. Here we review current evidence and guidance on monitoring patients with FMF in clinical practice, and propose flexible recommendations for long-term monitoring in FMF, that can be tailored to the needs and setting of the individual clinic.

**Methods:**

In this expert-informed narrative review based on a systematic literature search, published evidence was used to inform and augment the clinical experience and expert opinion of a group of international experts in FMF.

**Results:**

A total of 42 publications were included for review, including several treatment guidelines for FMF as well as publications relating to clinical biomarkers (primarily acute phase reactants), patient-reported outcomes and various disease scales and scores. While much of the guidance from these publications is broadly consistent, with agreement on core monitoring principles and parameters, the lack of validated, evidence-based recommendations highlights the need for flexibility in clinical practice.

**Conclusion:**

This review provides a comprehensive schedule of parameters with suggested monitoring frequencies, centred around three key principles of FMF monitoring: assessing disease characteristics, monitoring for subclinical inflammation, and assessing the tolerability of current medication and possible side effects. It is our hope that this will prove a useful starting point for creation of tailored monitoring schedules by clinics based on their specific resources, patient needs, and clinical priorities.

## 1 Introduction

Familial Mediterranean fever (FMF) is a hereditary autoinflammatory syndrome affecting 120–150,000 people globally, predominantly Turks, Armenians, Levantine Arabs and non-Ashkenazi Jews (1). Caused by *MEFV* gene mutations, FMF is characterised by recurrent episodes of fever, arthritis, serosal inflammation, myalgia, and erysipelas-like erythema (2–4). FMF negatively impacts health-related quality of life (QoL) and is associated with fibromyalgia, depression, anxiety, poor sleep, and fatigue (5–7). Frequent attacks and chronic inflammation can cause complications such as anaemia, growth retardation, delayed puberty, infertility, amyloid A (AA) amyloidosis, chronic kidney disease and intra-abdominal manifestations (4, 8). Inflammation is the main determinant of long-term prognosis and the only modifiable risk factor for adverse outcomes (9, 10).

FMF’s most serious complication is systemic AA amyloidosis (11). Renal failure resulting from amyloidosis is the major cause of death in FMF, occurring in up to 60% of untreated patients (12, 13), especially Turks, Armenians and *M694V* homozygotes (14, 15).

Colchicine, the standard treatment for FMF for over 50 years, is effective in preventing attacks and amyloidosis (16), but only with strict adherence (17). Approximately 5% of patients are non-responsive to colchicine (18), and many have subclinical inflammation while using colchicine (19). Since 2007, interleukin-1 (IL-1) inhibitors have been increasingly and successfully used in cases of insufficient response, toxicity or intolerance to colchicine (20, 21).

The ultimate goal of FMF treatment is the complete resolution of untriggered attacks, normalising subclinical inflammation between attacks and preventing long-term complications (17, 22). A ‘treat-to-target’ approach to therapy is increasingly recommended (17, 23). This involves adjusting therapy based on pre-defined treatment targets (23), and requires effective monitoring to assess the impact of therapeutic strategies (24, 25). Monitoring is also important to ensure that amyloidosis does not develop unchecked (22), and may also help support adherence by identifying issues affecting the patient’s commitment to taking prescribed treatment.

This publication aims to review current evidence and guidance on monitoring FMF patients in clinical practice, including circumstances under which more frequent monitoring may be required. Based on our findings, we propose flexible recommendations for long-term monitoring in FMF for clinics to use when developing their own management protocols.

## 2 Methods

This was an expert-informed narrative review based on a systematic literature search of the PubMed database, performed according to the parameters shown in **Table 1**. No field tags or additional PubMed filters were applied within the search string. The search strategy and eligibility criteria were defined before screening; the review protocol was not registered. Resulting publications were screened by title and abstract by a single reviewer for relevance and according to predefined exclusion criteria. Publications prior to 2016 were excluded during screening as this timepoint marked the publication of the European League Against Rheumatism (EULAR) guidelines for management of FMF, upon which this review builds (22). This timepoint also represents a milestone in understanding FMF: around this time, conceptualisation of the mechanism behind FMF shifted dramatically, changing from ‘loss-of-function mutations within a counter-inflammatory gene’ to ‘gain-of-function mutations within a pro-inflammatory gene’ (26). Non-English language publications were also excluded. Because this review aimed to capture a wide range of evidence and guidance relevant to monitoring patients with FMF, no further restrictions were applied based on study design, sample size, comparator, intervention type, or specific outcome measures. Publications were excluded for lack of relevance only if they did not address monitoring-related aspects of FMF.

**Table 1:** Literature search parameters.

Parameters	Search string	Excluded
Familial Mediterranean fever	(familial Mediterranean fever) AND ((biomarker*) OR (monitor*) OR (management) OR (guidelines) OR (treat-to-target) OR (wearable) OR (quality of life))	• Non-English language publications • Publication date prior to 2016 • Lack of relevance to monitoring patients with FMF
AND one or more of the secondary search terms combined with OR: • Biomarker/biomarkers • Monitor/monitoring • Management • Guidelines • Treat-to-target • Wearable • Quality of life		

FMF, familial Mediterranean fever.

Full texts of publications not excluded by the first screening round were examined in a second round by the same reviewer according to the same criteria. Those that remained eligible after full-text review were included. After completion of the formal literature search, the 2024 update of the EULAR/PReS endorsed recommendations for the management of FMF became available (27). Because of its direct relevance to current best practice, this guideline was reviewed separately and used to cross-check and refine the final expert monitoring framework. It was not included in the numerical summary of publications retrieved by the formal search.

Considering the evidence and recommendations from the retrieved literature and our own clinical experience, recommendations for monitoring were developed, going through several rounds of review and comment before a final version was agreed by all authors. The final monitoring schedule was developed by integrating recommendations from guidelines and consensus statements, evidence from observational and interventional studies, and expert clinical opinion. For transparency, items in the monitoring schedule are identified according to their primary source basis as guideline/consensus-supported, supported by limited clinical evidence (including observational, interventional or validation studies), or based primarily on expert opinion.

## 3 Results

**Figure 1:**
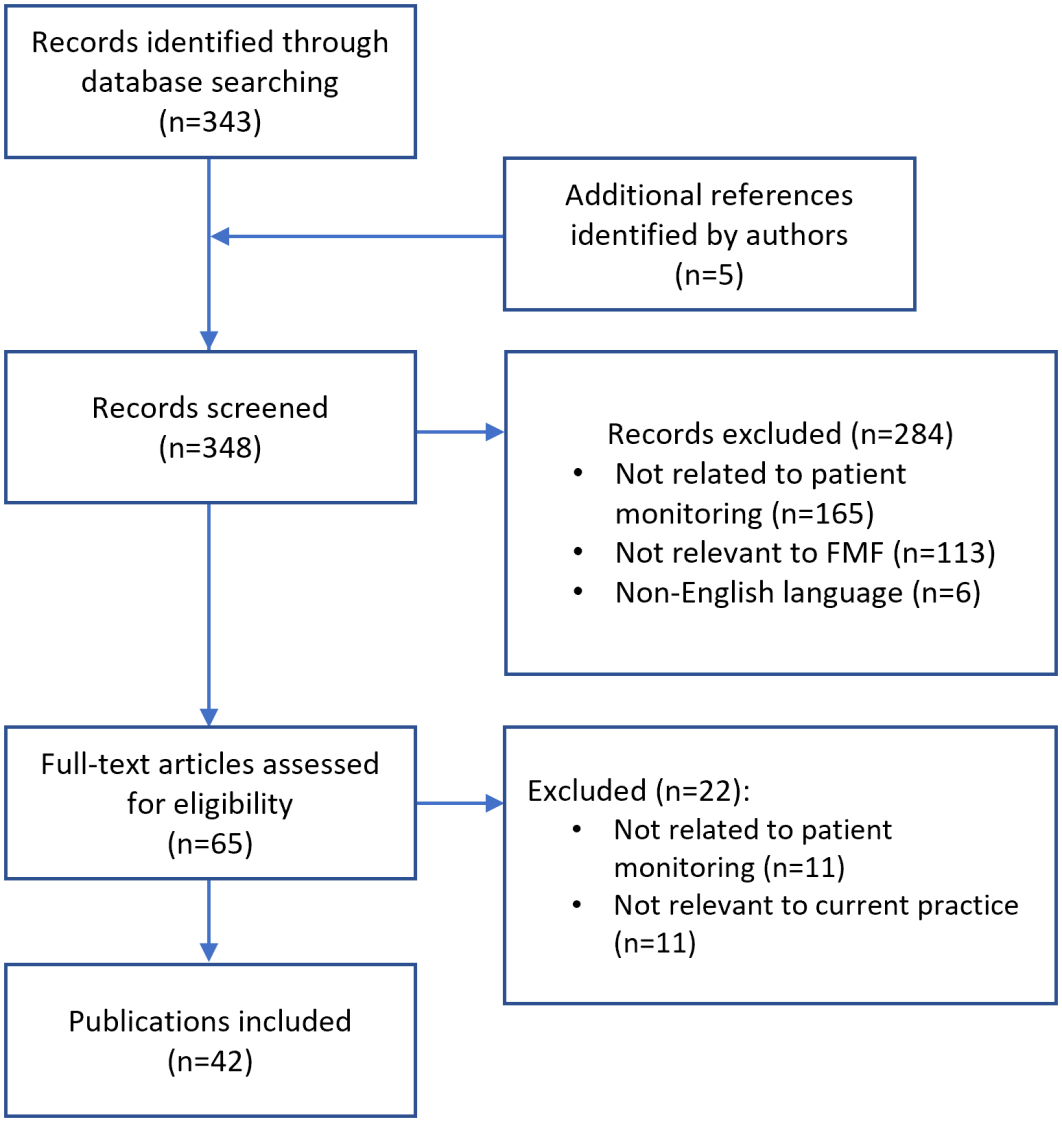
Literature review flow diagram.

A systematic literature search was performed on 9 April 2024 (**Figure 1**). From 343 publications initially retrieved, 42 publications were included for review. Of these, 7 were treatment guidelines, consensus statements or similar publications providing recommendations on FMF management relating to monitoring (10, 17, 22, 23, 28–30). Biomarkers of inflammation, disease activity, amyloidosis, or dosing requirements were the focus of 15 publications (11, 31–44), while 10 publications discussed QoL and other patient-reported outcomes (PROs) (6, 45–53). Scales and scores for monitoring were discussed in 7 publications (8, 24, 54–58), and three publications discussed adapting monitoring schedules according to different patient needs (59–61). There follows a summary of key information from these publications. Additional references not retrieved via the literature search are cited where needed to provide background context, or to identify the original sources for established concepts, scales and tools discussed in the retrieved publications.

### 3.1 Acute phase reactants

Acute phase reactants (APR) are plasma proteins produced primarily by the liver as a response to infection, inflammation, and trauma (33). In FMF, the main APR are C-reactive protein (CRP), serum amyloid-A (SAA), and fibrinogen (reflected by erythrocyte sedimentation rate [ESR]).

During an acute FMF attack, APR levels significantly increase (12, 62). Between attacks, measurements of CRP and SAA have revealed significant periods of subclinical inflammation in many patients (19). Chronic SAA elevation is considered essential for development of amyloidosis in FMF (33). The publications retrieved by the literature search covered these established biomarkers as well as more novel potential biomarkers, as summarised in **Table 2**.

**Table 2:** Summary of biomarkers discussed in relation to FMF monitoring.

Biomarker	Clinical significance in FMF	Strengths	Limitations	Current applicability in routine practice
CRP	Established APR used to assess inflammation during and between attacks; persistent elevation may suggest active disease or subclinical inflammation.	Widely available, cost-effective and familiar to clinicians; suitable for routine monitoring in many settings.	Non-specific; may be influenced by multiple non-FMF factors; may be less sensitive than SAA between attacks.	Routine clinical monitoring; interpret in clinical context.
SAA	Key APR for subclinical inflammation and amyloidosis risk.	More sensitive than CRP between attacks. Useful in high-risk patients and where subclinical inflammation or amyloidosis risk is suspected.	Not universally available or standardised.	Recommended where available, especially in high-risk patients; not mandatory where unavailable or unaffordable.
ESR/fibrinogen	Part of the acute phase response; complements CRP/SAA assessment.	Widely available and inexpensive; useful as an adjunctive inflammatory measure.	Indirect and non-specific.	Routine or adjunctive clinical monitoring depending on local practice.
S100 proteins	Investigated as inflammatory biomarkers in FMF and other autoinflammatory diseases.	May capture inflammatory activity not fully reflected by CRP/SAA.	Evidence is inconsistent; incremental value over established APR is unclear.	Research use only at present; not recommended for routine monitoring.
Other emerging biomarkers (YKL-40, pentraxin-3, sTREM-1, uNGAL)	Investigated as markers of chronic inflammation, amyloidosis risk, or early renal damage.	May provide additional information on inflammation or organ involvement beyond conventional APR.	Evidence is limited, based on small studies, and clinical utility remains uncertain.	Research use only; not recommended for routine monitoring.

APR, acute phase reactant; CRP, C-reactive protein; ESR, erythrocyte sedimentation rate; FMF, familial Mediterranean fever; SAA, serum amyloid A; sTREM-1, soluble triggering receptor expressed on myeloid cells-1; uNGAL, urinary neutrophil gelatinase-associated lipocalin.

#### 3.1.1 SAA and CRP

An analysis of 570 children with FMF found significantly higher CRP levels during attacks than between attacks, and in patients with homozygous *M694V* mutations compared to other mutations (38). Strong correlations between SAA and CRP during FMF attacks suggest it may be sufficient to measure CRP alone during this phase (33). However, between attacks, SAA appears more sensitive than CRP, and the correlation between the two, though still significant, is weaker (11).

A CRP threshold of <5 mg/L in children and <8.75 mg/L in adults corresponds well to an SAA level of <10 mg/L (a level associated with lower mortality and better renal prognosis) in attack-free periods (41). However, prednisone use alters the relationship between SAA and CRP, suggesting caution is needed when using CRP to predict SAA levels in patients receiving steroids (11).

#### 3.1.2 S100 proteins

Recent investigation of the phagocyte-specific S100 proteins has revealed that levels of S100A8/A9 and S100A12 can potentially be used to differentiate systemic juvenile idiopathic arthritis (JIA) from systemic infections (37). Levels of S100A8/A9 are much greater in FMF patients than healthy controls, even between attacks (42). One study reported significantly higher levels of S100A12 in FMF patients than healthy controls, but no significant differences between attacks and attack-free periods (35). In contrast, another study reported higher S100A12 levels in FMF patients during attacks compared to attack-free periods, as well as between FMF patients and controls (43). However, the differences were more pronounced for CRP and ESR, raising questions about the clinical relevance of these findings.

Combining two parameters, particularly attack frequency and S100A12 or S100A8/9, was shown in one study to better predict the need for future colchicine dose increase (39). However, it is unclear whether investigators measured S100 proteins during attacks or in attack-free periods, a critical distinction for clinical applications. Overall, S100A8/A9 and S100A12 remain investigational biomarkers in FMF.

#### 3.1.3 Other biomarkers

A study examining levels of SAA and two novel biomarkers of chronic inflammation, YKL-40 and pentraxin-3, found a correlation between SAA and YKL-40 in FMF patients. YKL-40 had slightly higher positive predictive values than SAA; however, this biomarker’s clinical relevance is limited due to lack of capacity for its measurement (34).

The serum soluble form of triggering receptor expressed on myeloid cells-1 (sTREM-1) has been investigated as a biomarker of amyloidosis (36). Levels of sTREM-1 were significantly higher in FMF patients with amyloidosis versus those without, even when SAA levels were normal. However, study limitations include disparities between the amyloidosis and non-amyloidosis groups in overall patient numbers and proportions of patients under anti-IL-1 therapy. Also, no patients in the amyloidosis group had an attack during the study, compared to 22% of the non-amyloidosis group. Conclusions must be therefore drawn with caution.

Urine neutrophil gelatinase-associated lipocalin (uNGAL) may have a role as an early biomarker of renal damage in FMF according to a small pilot study, but larger studies are needed to confirm the findings (40).

### 3.2 Scales and scores of disease activity, damage and severity

#### 3.2.1 Autoinflammatory disease activity index

The AIDAI is a disease activity scoring system for several auto-inflammatory diseases (AIDs), including FMF (24). It was developed to provide a common definition of disease activity and standardised disease activity score (63), and an updated version was validated several years later (64).

Based on PROs, the AIDAI is composed of 12 items: fever, overall symptoms, nausea/vomiting, abdominal pain, diarrhoea, chest pain, painful nodes, arthralgia/myalgia, joint swelling, headaches, eye manifestations and skin rash (29). It has demonstrated high sensitivity and specificity in defining patients with active AIDs (64).

The AIDAI was included in several publications retrieved by the literature search, including a real-world study of 101 patients with FMF on the impact of anti-IL-1 therapy on disease activity which found the AIDAI valuable to standardise assessment and objectively evaluate drug efficacy (54). It was also used in the phase III CLUSTER study of canakinumab, to record disease activity in 173 patients with AIDs (60 with active colchicine-resistant FMF) (24). A cut-off score of 12 for inactive disease calculated for FMF demonstrated a sensitivity and specificity of 75% and 80%, respectively (24).

Some treatment guidelines recommend the AIDAI to help define refractory FMF, guide management, and standardise clinical trial outcome measures (29, 30). However, its limitations include the need for long-term diary completion (57), which many patients find challenging. Also, consistently attributing general symptoms (such as arthralgia, myalgia, headaches, or ocular symptoms) to FMF could lead to overestimation of disease activity.

#### 3.2.2 Autoinflammatory disease damage index

The ADDI was developed to systematically assess long-term organ damage and other complications of chronic inflammation in AID including FMF (65, 66). It comprises 18 items grouped into 8 categories (reproductive, renal/amyloidosis, developmental, serosal, neurological, ears, ocular and musculoskeletal) scored according to the presence of damage, defined as persistent or irreversible change in structure or function present for at least 6 months (65). The ADDI was envisaged as one of several measures of disease burden to include metrics of disease activity, disease severity and QoL (65), and its use was recommended in a 2017 review of FMF management (29). Another publication defined a modified version of the ADDI, excluding chronic musculoskeletal pain. Patients were stratified according to presence or absence of damage defined by the ADDI and modified ADDI. Factors linked to a severe FMF phenotype (*M694V* homozygous mutation and persistent inflammation) were predictors of the modified ADDI score only (32). The modified ADDI has subsequently been used in a study measuring the efficacy of IL-1 inhibitors in preventing damage, as the investigators deemed it more appropriate than the ADDI for this purpose, considering the subjective nature of musculoskeletal pain (44).

#### 3.2.3 International severity scoring system for FMF

Inconsistencies between existing disease severity scores for use in children with FMF prompted development of a new evidence-based severity assessment tool, the ISSF (67), created and validated by an international consortium of FMF experts (8).

The ISSF is intended to permit simple and uniform assessment of FMF disease severity, guiding the intensity of therapy required (8). A study utilising the ISSF to evaluate disease severity in 150 patients concluded that it is a user-friendly tool that can help identify those at high risk of severe disease (61).

### 3.3 Measures of QoL and other PROs in FMF

Various standard measures are used to evaluate QoL in patients with FMF (58). In the retrieved publications, the Nottingham Health Profile was used to measure QoL in conjunction with measures of physical activity and mobility in adults with FMF (50), while the Pittsburgh Sleep Quality Index (PSQI), Multidimensional Assessment of Fatigue, and Hospital Anxiety and Depression Scale (HADS) were used to investigate the association between sleep quality and disease activity (6). Short-form 36 (SF-36) was used to monitor the impact of IL-1 therapy on QoL in one study (52), while in another it was used with the Work Productivity and Activity Impairment Specific Health Problem v2.0 to assess QoL and work productivity in FMF patients (49).

Several reports focussed on measuring QoL in children with FMF, including the Kinder Lebensqualität Fragebogen questionnaire (53) and the Pediatric QoL (PedsQL) Inventory 4.0 (48). The PedsQL Multidimensional Fatigue Scale (PedsQL-MFS) and the PSQI were used to assess fatigue and sleep quality in children with FMF (46). The PedsQL-MFS was validated for assessment of fatigue in adolescents with FMF, with excellent reliability and internal consistency (47). However, translated versions of these instruments across various languages are not always validated, which may impact their reliability in different populations.

In 2021, a specific QoL scale for patients with FMF was developed and validated. The FMF-QoL scale includes 20 questions assessing the impact of physical, social and recreational, psychological, and sleep factors on patient health status (51). It significantly correlated with other QoL measures, including physical and mental aspects of the SF-36 and anxiety and depression items of HADS, and investigators concluded that it was valid and simple to administer (45).

### 3.4 Factors affecting monitoring requirements

Several publications described circumstances in which monitoring might need adjusting according to the needs of the patient. *M694V* homozygous patients are at increased risk of amyloidosis (31), so may warrant more frequent monitoring, as may patients who do not meet Eurofever/PRINTO classification criteria (EPCC), which has been associated with a lack of response to colchicine (59). Similarly, patients meeting the EPCC including genetic variables, with a confirmed pathogenic *MEFV* variant, may require different monitoring than those meeting the criteria based only on clinical variables (i.e. without a confirmed pathogenic variant or with a variant that does not meet the EPCC). They may have earlier disease onset and more frequent attacks, and therefore need closer follow-up (60).

Some patients may have increased APR due to factors other than FMF (e.g. infectious, autoimmune or chronic metabolic diseases, obesity, cancer, drugs, smoking, alcohol abuse) (34), suggesting additional measures may be needed to rule out subclinical inflammation. Frequent monitoring is required for patients with hepatic or renal impairment, repeatedly elevated APR or unstable disease, pregnancy, or at any time when the dose is adjusted or toxicity develops (17, 22). More frequent evaluations may also be needed to adjust treatment in children, in whom frequent blood sampling may be impractical (22).

### 3.5 Published recommendations on monitoring

**Figure 2:**
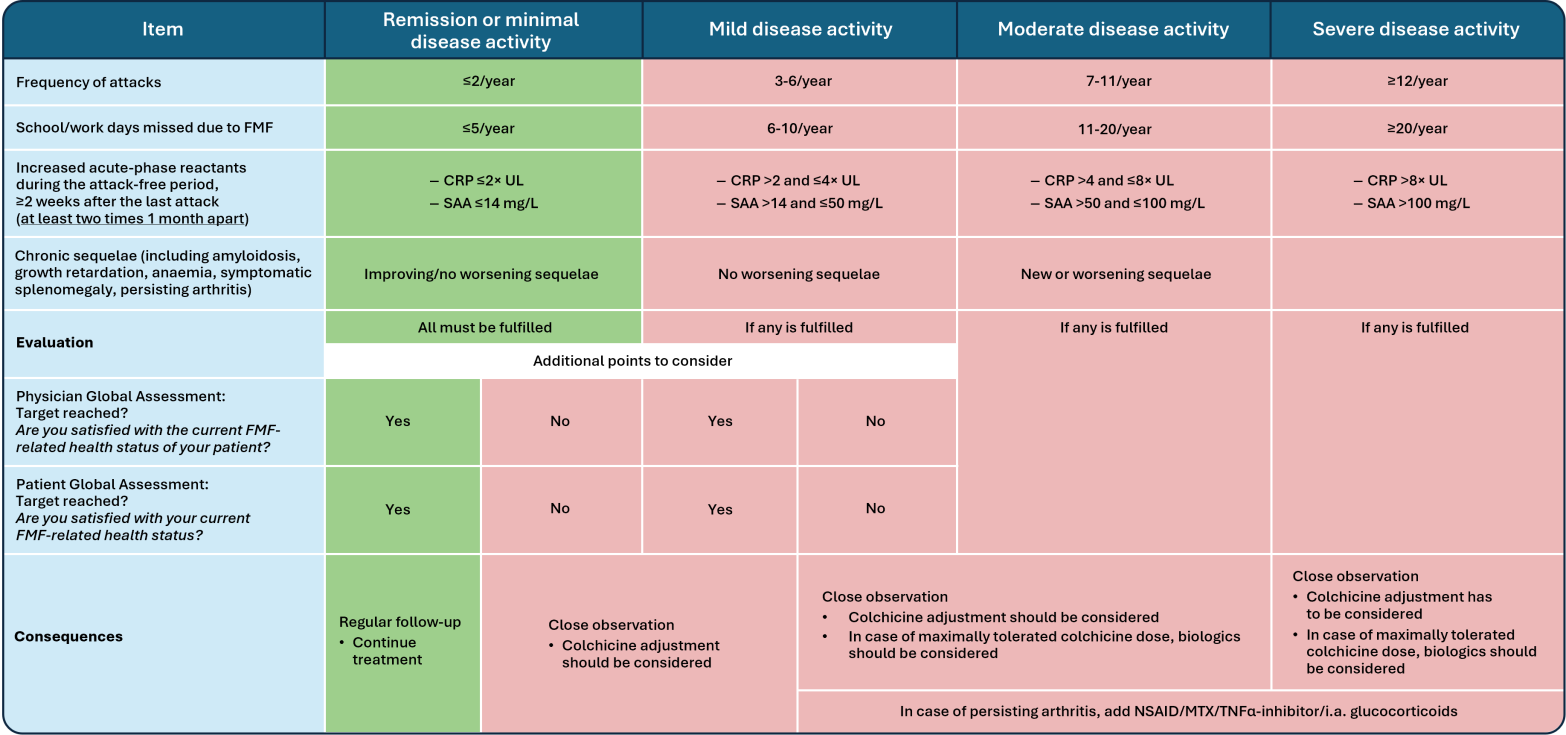
Composite score of multidimensional treatment targets to assess disease activity in children with FMF according to PRO-KIND treat-to-target initiative (23) CRP, C-reactive protein; FMF, familial Mediterranean fever; MTX, methotrexate; NSAID, non-steroidal anti-inflammatory drug; UL, upper limit; SAA, serum amyloid A. Adapted from Ehlers et al., (23) and shared under a Creative Commons Attribution 4.0 International License, available at http://creativecommons.org/licenses/by/4.0/.

Published guidance relevant to FMF monitoring includes the 2016 EULAR recommendations for FMF management (22), updated in 2024 with the publication of the EULAR/PReS endorsed recommendations (27), and French national guidelines emphasizing a treat-to-target approach (17). Other relevant publications include the German Protokolle in der Kinderrheumatologie (PRO-KIND) initiative, which proposed a multidimensional treatment target for children (**Figure 2**) (23), Turkish paediatric consensus statements on FMF follow-up (28), an international modified Delphi consensus on colchicine resistance/intolerance in FMF which included guidance on PROs to guide disease management (30), and a review by Tufan and Lachmann providing FMF treatment and monitoring recommendations (10).

Much of the guidance provided by these publications is in broad agreement, with consistency on core monitoring principles and parameters. Most guidelines concur that follow-up visits should take place every 3–6 months for newly diagnosed or uncontrolled patients (10, 17, 22, 23, 28). The 2024 EULAR/PReS update similarly recommends regular monitoring, which should be more frequent at diagnosis and when disease is uncontrolled (27). The 2024 EULAR/PReS update also recommends close monitoring, ideally every 3 months, when biologic doses are tapered or administration intervals are increased, because of the risk of flare-ups or worsening inflammatory findings (27). In patients stable on treatment, the 2016 EULAR and French guidelines recommend visits every 6–12 months (17, 22), while the 2024 EULAR/PReS update states that 6-monthly visits may suffice when disease is controlled (27), similar to recommendations by the PRO-KIND initiative and Tufan and Lachmann (10, 23). The latter also suggest the use of physical activity trackers for attack detection between clinic visits (10), although this use should currently be regarded as exploratory.

Across guidelines, regular monitoring of CRP and/or SAA is recommended to assess inflammation. The 2024 EULAR/PReS update identifies complete control of subclinical inflammation as a core treatment goal and includes biomarkers such as CRP and SAA in its proposed minimum outcome set (27). Due to its accessibility and cost-effectiveness, CRP is generally recommended over SAA, other than in high-risk cases requiring the increased sensitivity of SAA (17, 22, 28). Notably, the 2024 EULAR/PReS update recommends the preferential use of SAA and proteinuria for monitoring subclinical inflammation in patients with AA amyloidosis where possible, subject to SAA availability and cost (27). Other routinely recommended laboratory tests include haemogram/complete blood counts, liver enzymes and kidney function tests. Urinalysis including protein/creatinine ratio is universally recommended for amyloidosis screening (10, 17, 22, 28).

Several guidelines recommend the AIDAI for evaluating treatment response (22, 28, 30), while the PRO-KIND initiative combines aspects of the AIDAI and ISSF to create their multidimensional treatment target. Other PROs suggested to help guide disease management include restrictions in daily activity, fatigue and chronic pain, missed work/school days, juvenile autoinflammatory disease multidimensional assessment report (JAIMAR) and generic QoL measures (30). The 2024 EULAR/PReS update further reinforces patient-centred assessment by proposing a minimum outcome set that, in addition to biomarkers, includes measures of clinical disease activity such as attack frequency and physician assessment, and patient-reported outcome and experience measures (27).

The importance of adherence to therapy is emphasised in recent guidance. The 2024 EULAR/PReS update supports regular adherence monitoring and refers to a dedicated EULAR publication on points to consider for screening, monitoring and managing non-adherence in people with rheumatic and musculoskeletal diseases (27, 68). Suggested approaches include the use of questionnaires, pharmacy indicators, drug levels, and diaries, although the authors stress that these should not replace open discussion of adherence with the patient in a safe, blame-free environment (68). Validated adherence assessment tools for FMF include the Medication Adherence Scale for Familial Mediterranean Fever (MASIF), developed for use in children with FMF (23, 69).

In addition to assessing treatment adherence, monitoring for adverse events (AEs) such as digestive symptoms is recommended in colchicine-treated patients (17, 22, 28). When treating with IL-1 inhibitors, the French guidelines recommend regular testing for gamma-glutamyl transferase (GGT), cholesterol and triglycerides (17), while the Turkish consensus highlights injection-site tolerability as a potential AE related to IL-1 inhibitor therapy (28).

## 4 Discussion

Due to the rare nature of FMF, current management guidelines have not been validated in real-world settings, with most recommendations based on expert opinion due to an insufficiency of robust evidence in the literature. In the absence of such evidence, an expectation of rigid adherence to guidelines may be unreasonable. For this reason, the recommendations we provide here, according to our own expert opinion as well as our review of the current scientific literature, are intended as flexible suggestions which we envisage will help healthcare providers to develop tailored monitoring schemes adapted according to their patient population, clinic’s capabilities, and local healthcare systems.

**Figure 3:**
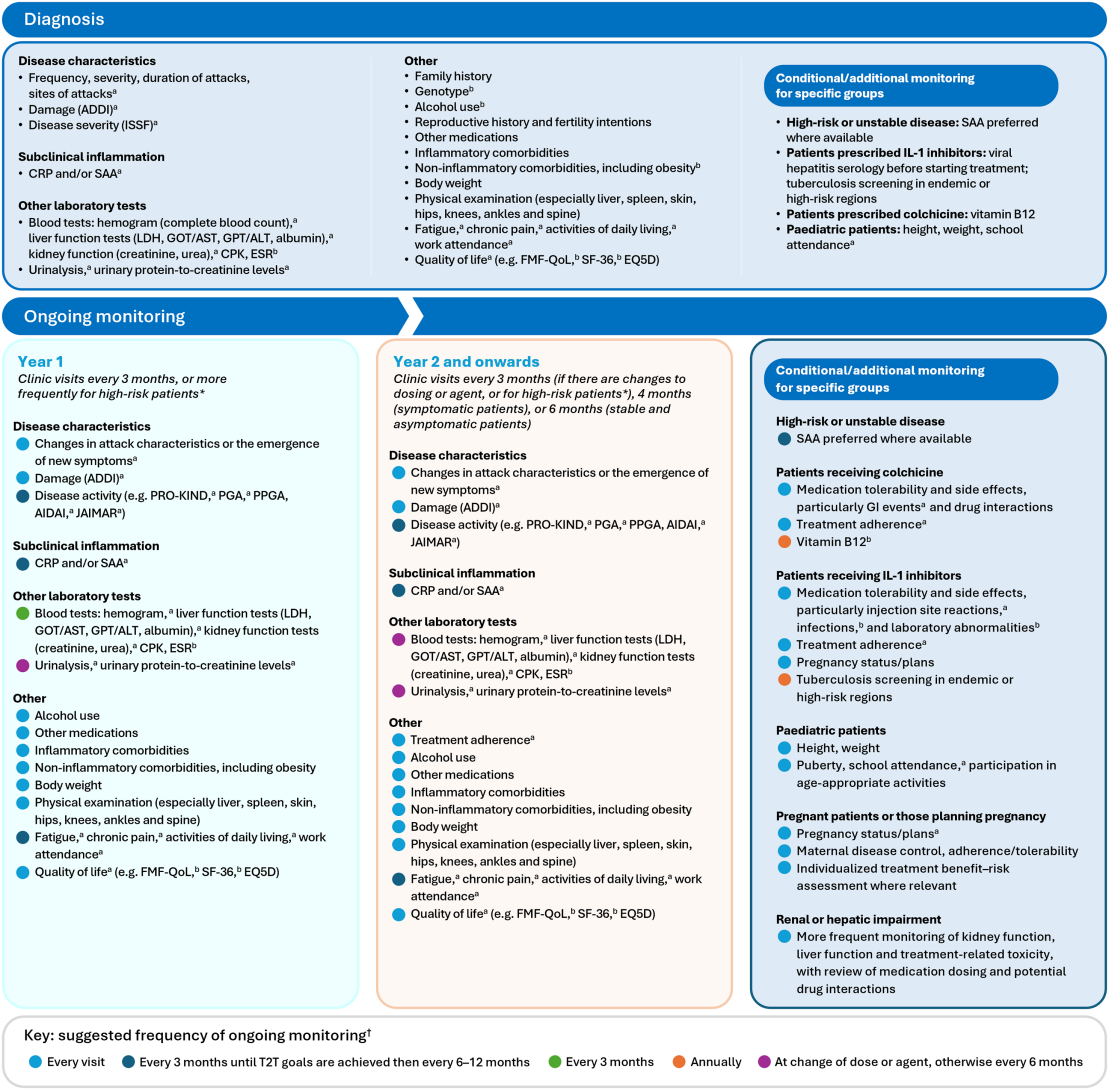
Flexible monitoring schedule for FMF: suggested parameters and frequencies for tailored clinical application. Superscript letters indicate the source basis for inclusion in the monitoring framework: a, supported by published guideline or consensus recommendations; b, supported by limited clinical evidence, including observational, interventional or validation studies. Parameters without a superscript letter are expert opinion recommendations. The source basis applies to inclusion of the parameter, not necessarily to the proposed monitoring frequency. *Frequency of assessment can be more often in high-risk individuals. High-risk individuals for adverse outcomes include those with a high ISSF score, pre-existing damage, prior amyloidosis or proteinuria, persistent APR elevation, poor adherence, colchicine intolerance or resistance, a family history of amyloidosis, homozygous or compound heterozygous exon 10 variants at positions 680–694 amino acids, pregnancy, diagnosis of protracted febrile myalgia at a young age and liver or kidney dysfunction resulting from any cause. ^†^Suggested clinic-visit frequencies are informed by published guideline/consensus recommendations, whereas the timing of individual monitoring parameters reflects expert interpretation of the available guidance and evidence and should be adapted according to clinical context (e.g. disease stability, treatment, age, pregnancy status, comorbidities, local resources and patient risk). ADDI, autoinflammatory disease damage index; AIDAI, auto-inflammatory diseases activity index; ALT, alanine transaminase; AST, aspartate aminotransferase; CPK, creatine phosphokinase; CRP, C-reactive protein; EQ5D, EuroQol five-dimensional instrument; FMF, familial Mediterranean fever; GOT, glutamate oxaloacetate transaminase; GPT, glutamate pyruvate transaminase; IBD, inflammatory bowel disease; ISSF, International severity score for FMF; JAIMAR, juvenile autoinflammatory disease multidimensional assessment report; LDH, lactate dehydrogenase; PGA, physician global assessment of disease activity; PPGA, patient parent global assessment of disease activity; SAA, serum amyloid A; SF-36, short form 36; SpA, spondyloarthritis; T2T, treat to target.

A comprehensive schedule with suggested monitoring frequencies is provided in **Figure 3**, which includes recommended parameters to assess patients with FMF, both at diagnosis and during follow-up, including during treatment with colchicine and/or IL-1 inhibitors. Suggested monitoring frequencies reflect a combination of published guideline recommendations, available evidence and expert consensus. Where published guidance supports monitoring but does not specify an exact interval, the proposed frequency represents our interpretation of best practice in light of the available guidance and evidence. We expect this schedule to be adapted by clinics based on their resources, patient needs, and clinical priorities. However, at a minimum, any schedule should address three key principles of FMF monitoring at every visit: 1) Assess disease characteristics; 2) Monitor for subclinical inflammation; and 3) Assess tolerability of current medication and possible side-effects.

### 4.1 Disease characteristics

At diagnosis, the frequency, severity, duration and sites of attacks should be recorded, with any changes or new symptoms tracked during follow-up. Special attention should be paid to flare-ups during menstruation in adolescent and adult female patients, with patients encouraged to keep disease activity diaries to record this information. Communicating the importance of accurate information for disease management may help to motivate patients to maintain such records.

Disease-related damage should be assessed at diagnosis, and new damage or worsening tracked during follow-up. The ADDI is recommended for this purpose, since it is a validated instrument, but some clinicians may prefer to use the modified ADDI due to its improved specificity. Severity of disease may be assessed at diagnosis using the ISSF. Several scales are available to measure disease activity and functional status, with the PRO-KIND composite score (**Figure 2**), PGA, PPGA and AIDAI recommended as useful options. JAIMAR may also be valuable in younger patients. Consistent use of the same scale with a patient allows changes to be tracked over time.

### 4.2 Subclinical inflammation

In patients with suspected periodic or recurrent fever syndromes, persistent elevated APR levels can be a diagnostic clue indicative of FMF. In those diagnosed with FMF, regular monitoring for subclinical inflammation is crucial, as it predicts poor outcomes even in patients who appear to be symptom-free. Testing between attacks is more informative than measuring levels during attacks, and should be performed at least 2 weeks after any febrile episode.

CRP and SAA are the main biomarkers for subclinical inflammation in FMF. CRP levels should normalise during remission. Elevated levels (>5 mg/L) without other explanations (e.g. acute infection), especially if accompanied by normocytic anaemia, are suggestive of active disease. CRP is widely available and cost-effective, and is therefore suitable for routine monitoring in many settings. However, CRP may be influenced by infection, obesity, corticosteroid use, and other inflammatory or metabolic conditions, so elevated CRP should be interpreted in clinical context. SAA can be used instead of or in addition to CRP, and may be more sensitive than CRP for detecting subclinical inflammation between attacks. Where available, we recommend measurement of SAA in symptom-free intervals at least once a year, particularly in high-risk patients, those with suspected subclinical inflammation, persistent or unexplained APR elevation, prior amyloidosis or proteinuria, or other risk factors for amyloidosis. However, SAA availability, assay standardisation, cost and interpretation vary between settings; therefore, use of SAA should be regarded as conditional on availability rather than mandatory. These practical constraints are particularly relevant in low-resource settings, including regions where FMF prevalence may be high; in such contexts, CRP, ESR and assessment of proteinuria may remain the most feasible routine monitoring tools.

Other inflammatory biomarkers discussed in the retrieved literature, such as S100 proteins, are not widely utilised outside a research setting, and are therefore not yet recommended for routine monitoring, although this may change with emerging evidence. While cytokine-based monitoring was not a focus of the literature retrieved by our search, cytokines and inflammasome-associated mediators, including IL-1β and IL-6, which are central to FMF pathogenesis, and IL-8, IL-22 and IL-23, whose plasma concentrations have been reported to be substantially higher in patients with FMF than in controls, remain an area of active research as potential indicators of disease activity and subclinical inflammation (70). Some of these mediators, especially IL-1β, are technically challenging to measure because of rapid degradation in blood samples. In practice, cytokine assays are not currently used in routine FMF monitoring, and further validation, standardisation and evidence of incremental value over established APR are required before they can be recommended for clinical monitoring.

### 4.3 Medication tolerability and side effects

Patients should be monitored for potential safety and tolerability issues. Colchicine intolerance is most often associated with gastrointestinal AEs (30). It can be helpful to educate patients and their families on safe practices related to colchicine therapy, such as avoiding drug interactions (cytochrome P450 3A4 and/or P-glycoprotein inhibitors, such as macrolide antibiotics, grapefruit juice, and certain azole anti-fungals) to prevent adverse effects. In patients with gastrointestinal symptoms, published guidance suggests practical measures such as dose splitting, temporary reduction of dairy products in those with suspected lactose intolerance, dose reduction, and antidiarrhoeal or spasmolytic agents (10, 22, 30). Based on our clinical experience, patients may also be advised to consider the timing of dairy intake relative to colchicine dosing, or to trial lactose-free alternatives where dairy appears to exacerbate symptoms.

Monitoring in patients receiving IL-1 inhibitors should be adapted according to the specific agent used, dosing schedule, local licensing status and practice, comorbidities and regional infection risk. AEs of note for patients treated with IL-1 inhibitors include injection site reactions and increased susceptibility to streptococcal infections and upper respiratory tract infections (mostly mild), and also some laboratory abnormalities, such as neutropenia, thrombocytopenia and elevated liver enzymes (71). Although the IL-1 inhibitors used in FMF treatment differ in terms of half-life, dosing interval and tolerability profile, these AEs are broadly relevant across IL-1 inhibitor therapy. The guidelines reviewed here do not provide sufficient detail to justify separate monitoring recommendations for each agent in this framework. Therefore, the schedule presents IL-1 inhibitor monitoring at class level, with additional monitoring to be adapted to the individual agent and patient context.

### 4.4 Laboratory tests

Blood and urine tests are essential for monitoring inflammation, organ function, and potential treatment complications in patients with FMF, particularly when initiating therapy or after a change of dose or agent. Important tests include hemogram, liver enzyme levels, kidney function tests, creatine phosphokinase and ESR. Urinalysis and urinary protein-to-creatinine ratio testing should be performed regularly (generally once or twice per year) to detect renal amyloidosis. Since long-term colchicine treatment has been associated with reduced vitamin B12 absorption and, in rare cases, vitamin B12 deficiency (29), we recommend annual monitoring of vitamin B12 levels in patients receiving long-term colchicine, to enable supplementation if necessary.

### 4.5 Other monitoring parameters

Family history should be taken and genotype determined at the first visit. This is important, since risk calculation is largely based on family history (e.g. relatives with a history of renal amyloidosis) and genotyping (e.g. *M694V* homozygotes are associated with higher risk of colchicine resistance). In patients with suspected FMF rather than established disease, genetic testing should be considered when CRP remains abnormally elevated for more than two weeks after attack resolution, particularly in individuals with a familial or ethnic background consistent with FMF.

During the initial visit, clinicians should discuss treatment adherence, alcohol use, and other factors that may affect disease control or medication safety. Active medications should be monitored for potential drug interactions, which could lead to potentially fatal elevated colchicine levels. Where relevant, reproductive health should be discussed proactively, including reproductive history, fertility intentions, pregnancy planning, and pregnancy status. For patients receiving colchicine, counselling should address safety during conception, pregnancy and lactation, as well as adherence and tolerability, since concerns about medication exposure may affect treatment continuation. In patients receiving IL-1 inhibitors, pregnancy status and plans should be reviewed at every visit; where pregnancy occurs or is planned, monitoring should include careful review of maternal disease control and ongoing individualised benefit-risk assessment.

Both inflammatory and non-inflammatory comorbidities should be assessed. Spondyloarthritis, inflammatory bowel disease, vasculitis and inflammatory skin diseases may all be FMF-associated inflammatory comorbidities, needing concomitant management if present. Active infections should be assessed at every visit in IL-1-inhibitor-treated patients, given their immunosuppressive effect. Available evidence has not identified a link between IL-1 inhibitor therapy and viral hepatitis reactivation or exacerbation, although data are limited (72, 73). Viral hepatitis serology should be performed before starting IL-1 inhibition, with repeat testing thereafter required only in special cases (e.g. elevated liver enzymes/bilirubin or after blood transfusion). In IL-1-inhibitor-treated patients in endemic or high-risk regions, tuberculosis screening should be performed initially and then annually.

Physical examinations should focus on liver, spleen, skin, hips, knees, ankles and spine; paediatric patients should be assessed for height and weight gain at every visit. For patients receiving IL-1 inhibitors, weight should be assessed closely, so dose adjustments may take place in a timely manner, if needed. Excessive weight gain has occasionally been reported with IL-1 inhibitors (17, 54); patients with pre-existing metabolic disorders receiving these agents may warrant special attention (54).

Fatigue, chronic pain and general well-being should be assessed. Discussing restriction in daily activities can provide insights into social effects of FMF. In terms of work productivity, absenteeism and presenteeism are equally important. More formal measures of QoL may be useful, including the disease-specific FMF-QoL and non-disease-specific tools like the SF-36 or EuroQol five-dimensional instrument (EQ5D).

In paediatric patients, in addition to tracking changes in height and weight, monitoring should also address puberty, school absenteeism, participation in normal activities, and the impact of disease on family functioning. Education of parents or caregivers, alongside age-appropriate education of the child, is important to support adherence, recognition of attacks, and timely reporting of adverse effects. Monitoring schedules should also account for the practical and emotional challenges of repeated blood sampling in children.

### 4.6 Monitoring frequency

Clinic visits should be most frequent at diagnosis, and whenever changes are made to the patient’s regimen, including if attempting therapy tapering. Three-monthly visits may be appropriate for most patients at this stage, but more frequent monitoring may be necessary for those at high risk for adverse outcomes, including those with high ISSF, pre-existing damage, prior amyloidosis or proteinuria, persistent APR elevation, poor adherence, colchicine intolerance or resistance, family history of amyloidosis, homozygous or compound heterozygous exon 10 variants at positions 680-694, pregnancy, diagnosis of protracted febrile myalgia at a young age or any liver or kidney dysfunction.

If patients remain stable and asymptomatic, visits can be reduced in frequency to 6-monthly. In generally stable but symptomatic patients, visits every 3 to 4 months may be required.

## 5 Conclusions

‘Treat-to-target’ goals in FMF aim to prevent flare-ups, maintain QoL and productivity, and minimise subclinical inflammation to avoid permanent damage. Appropriate monitoring is key to achieving these goals. Since evidence regarding the content and frequency of monitoring parameters is not robust enough for strict recommendations, institutions should develop their own monitoring protocols, based on the options available to them. Current guidance supports the individualisation of FMF monitoring according to clinical phenotype, genotype, inflammatory burden, treatment response and adherence. Future work should aim to determine whether emerging molecular and immunological biomarkers can support more personalised monitoring, provide added value over established monitoring approaches, and be feasibly integrated into routine clinical practice. In the meantime, we present here a recommended schedule of parameters to guide assessment at diagnosis and during follow-up, to help healthcare providers tailor their own monitoring protocols for FMF.
